# Efficient Generation of Lens Progenitor Cells from Cataract Patient–Specific Induced Pluripotent Stem Cells

**DOI:** 10.1371/journal.pone.0032612

**Published:** 2012-03-05

**Authors:** Xiaodi Qiu, Jin Yang, Tianjin Liu, Yongxiang Jiang, Qihua Le, Yi Lu

**Affiliations:** 1 Department of Ophthalmology, Eye and ENT Hospital of Fudan University, Shanghai, People's Republic of China; 2 Laboratory of Molecular Cell Biology, Institute of Biochemistry and Cell Biology, Shanghai Institutes for Biological Sciences, Chinese Academy for Sciences, Shanghai, People's Republic of China; The Walter and Eliza Hall of Medical Research, Australia

## Abstract

The development of a technique to induce the transformation of somatic cells to a pluripotent state via the ectopic expression of defined transcription factors was a transformational event in the field of regenerative medicine. The development of this technique also impacted ophthalmology, as patient-specific induced pluripotent stemcells (iPSCs) may be useful resources for some ophthalmological diseases. The lens is a key refractive element in the eye that focuses images of the visual world onto the retina. To establish a new model for drug screening to treat lens diseases and investigating lens aging and development, we examined whether human lens epithelial cells (HLECs) could be induced into iPSCs and if lens-specific differentiation of these cells could be achieved under defined chemical conditions. We first efficiently reprogrammed HLECs from age-related cataract patients to iPSCs with OCT-4, SOX-2, and KLF-4. The resulting HLEC-derived iPS (HLE-iPS) colonies were indistinguishable from human ES cells with respect to morphology, gene expression, pluripotent marker expression and their ability to generate all embryonic germ-cell layers. Next, we performed a 3-step induction procedure: HLE-iPS cells were differentiated into large numbers of lens progenitor-like cells with defined factors (Noggin, BMP and FGF2), and we determined that these cells expressed lens-specific markers (PAX6, SOX2, SIX3, CRYAB, CRYAA, BFSP1, and MIP). In addition, HLE-iPS-derived lens cells exhibited reduced expression of epithelial mesenchymal transition (EMT) markers compared with human embryonic stem cells (hESCs) and fibroblast-derived iPSCs. Our study describes a highly efficient procedure for generating lens progenitor cells from cataract patient HLEC-derived iPSCs. These patient-derived pluripotent cells provide a valuable model for studying the developmental and molecular biological mechanisms that underlie cell determination in lens development and cataract pathophysiology.

## Introduction

Age-related cataracts are one of the most prevalent ocular conditions and are responsible for nearly half of the cases of blindness worldwide [1]. The pathogenesis of cataracts is complex and involves both genetic and environmental factors. In contrast to the cellular and molecular complexities of most ocular tissues, the lens is a relatively simple structure. The lens is one of the most promising tissues for aging studies, due to the ease of obtaining lens epithelial and fiber cells, as well as the relative molecular simplicity of fully differentiated fiber cells [2]. Additionally, the lens is as accessible system for examining the fundamental aspects of embryonic induction [3]. Developmental defects in the lens are major causes of blindness and visual impairment among children. Because many of the pathways required for lens formation are also important for lens maintenance, a detailed understanding of lens development will provide a rational basis for the treatment of childhood cataract and may shed light on the lens-associated diseases observed during the aging process.

However, a systematic approach for studying human cataracts has been hampered by the lack of appropriate human-derived models and limitations of human primary lens culture [4]. One possible method for circumventing these issues is to induce human ES cells (hESCs) to differentiate toward lens progenitors and mature lens cells [5], [6]. The establishment of efficient differentiation procedures for the generation of lens cells from hESCs is an important step for understanding human embryonic lens development and related diseases. However, the use of human embryos is ethically controversial and may lead to tissue rejection, thereby hindering the potential application of hESCs. In addition, it is difficult to generate disease-specific ES cells, which are required for their effective application in clinical contexts. An alternative approach for the generation and study of pluripotent cells was recently described, which consisted of inducing a pluripotent status in somatic cells by direct reprogramming [7]. Induced pluripotent stem (iPS) cells generally exhibit a normal karyotype, are transcriptionally and epigenetically similar to embryonic stem (ES) cells and maintain the capacity to differentiate into derivatives of all three germ layers. The transplantation of different types of cells or tissues derived from iPS cells has recently become possible. The induction of pluripotency can be achieved by ectopically expressing factors known to be highly expressed in ES cells. Specifically, the transduction of four genes encoding the transcription factors OCT-4, SOX-2, C-Myc, and KLF-4 is generally used to reset the epigenetic and transcriptional status of somatic cells to those of pluripotent cells, which are functionally indistinguishable from ES cells [8], [9], [10], [11]. The application of this approach in human cells has enormous potential, allowing for the generation of patient-specific pluripotent stem cells for the study and amelioration of human diseases [12]. Somatic cell reprogramming has been performed using numerous somatic sources with variable kinetics and efficiencies [13], [14]. Therefore, it may be possible to efficiently generate lens progenitor cells from cataract patient-specific iPS cells that would aid studies concerning both lens development and mechanisms of cataractogenesis.

There are currently no patient-specific models for the mechanistic exploration of lens development and cataract formation. Furthermore, no research has been conducted to determine whether human lens epithelial cells (HLECs) from cataract patients may be induced into iPS cells or whether these iPSCs may subsequently be differentiated into lens progenitor cells under defined chemical conditions. To address these questions, we generated patient-specific iPS cells from lens epithelial cells. Next, we differentiated these iPSCs and ESCs into lens progenitor cells using a 3-stage procedure of sequential inhibition and activation of the FGF, TGF-β and Wnt signaling pathways. Additionally, we analyzed the expression of epithelial mesenchymal transition (EMT) markers, key lens regulatory genes and structural genes during lens development from iPS and ES cells. Our results demonstrate that patient–specific iPSCs can be used to generate lens cells efficiently.

## Materials and Methods

### Cell culture

Human lens epithelial cells were isolated from age-related cataract patients at the Eye and ENT Hospital of Fudan University. We thoroughly reviewed the patients' onset age, symptoms and family history. No evidence suggested that their cataracts were caused by congenital, traumatic or metabolic factors. The circular piece of the anterior capsule with the LECs attached was obtained by capsulotomy during cataract surgery and cultured directly without dispersion of the cells. After circular capsulorhexis, a section of the anterior capsule measuring approximately 5 mm in diameter was touched with an irrigation/aspiration tip and withdrawn from the eye by aspiration. The cells were cultured in Dulbecco's modified Eagle's medium (DMEM) (Gibco, Invitrogen, Grand Island, NY, USA) containing 15% fetal bovine serum (FBS) (Gibco, Invitrogen, Auckland, NZ). Skin biopsy samples were also obtained from these patients, digested with trypsin for 30 minutes and plated in a dish with DMEM+10% FBS Medium. 293T cells (ATCC, Manassas, VA, USA) were cultured in DMEM containing 10% fetal calf serum (FCS) [15]. The research followed the tenets of the Declaration of Helsinki, and written informed consent was obtained from the subjects following an explanation of the nature and possible consequences of the study. This study was approved by the Eye and ENT Hospital of Fudan University Ethical Committee (ID: KJ2010-41). Human H9 ES cells (Wicell) and iPSCs were cultured as previously described [16] and fully characterized using similar methodologies and criteria as described elsewhere.

### Plasmids and virus preparation

The lentiviral vectors obtained from Addgene were as follows: plenti-hOCT4, plenti-hSOX2 and plenti-hKLF4. The packaging plasmids (pCMV-gag-pol-PA and pCMV-VSVg) were generously provided by Dr. Trono of Geneva University. Lentivirus was collected 48 hours following cotransfection of the plasmids in 293T cells. Transfections were performed using Lipofectamine (Invitrogen, Carlsbad, CA, USA) in accordance with the manufacturer's recommendations.

### iPS cells generation

HLECs and human fibroblast cells were infected at passage 2. For viral transduction, 1×10^6^ HLECs and fibroblast cells were seeded on 60 mm gelatin-coated dishes. After the cells had spread evenly on the culture dishes, a solution of the three viruses was added to the medium with polybrene (Sigma, St. Louis, MO, USA, 8 µg/ml) for 48 hours. On the fifth day, the infected fibroblast cells were replated onto irradiated mouse embryonic fibroblasts (MEFs). After seven days, the DMEM medium was replaced with the standard human ES cell culture medium (Gibco, Invitrogen, Grand Island, NY, USA), and small colonies emerged in the culture dish. Single colonies were picked two days later and cultured until they grew into large colonies.

### Differentiation of iPS and ES cells

H9 human ES cells were cultured on MEFs in DMEM/F-12 (Gibco, Invitrogen, Grand Island, NY, USA) supplemented with ES medium. The culture medium was refreshed every two days, and the cells were passaged once a week. Cell differentiation was induced by the sequential administration of individual or specific combinations of growth factors using a 3-step induction procedure [6]: 1) administration of 100 ng/ml Noggin (R&D) from d 0 to 5; 2) a combination of 100 ng/ml bFGF (R&D), 20 ng/ml BMP4 (R&D) and 20 ng/ml BMP7 (R&D) from d 5 to 15; and 3) a combination of 100 ng/ml FGF2 (R&D) and 20 ng/ml Wnt-3a (R&D) from d 15 to 30.

### Real-time PCR

Total RNA was isolated using the Trizol Reagent (Invitrogen, Gaithersburg, MD, USA) and reverse-transcriped using the SuperScript II Reverse Transcriptase kit (Invitrogen, Carlsbad, CA, USA) according to the manufacturer's recommendations. Real-time PCR analysis was performed using the SYBR-Green PCR Master Mix (Applied Biosystems, Foster City, CA, USA). The expression levels of individual genes were normalized to ß-actin levels and are shown relative to the control samples as indicated. The primer sequences used in the real-time PCR assays are listed in Table 1.

**Table 1:** Primer sets for each human gene used in qRT-PCR assays.

Gene	Forward primer	Reverse primer
NANOG	5′-GGGCCTGAAGAAAACTATCCA-3′	5′-TGCTATTCTTCGGCCAGTTGT-3′
FGF-4	5′-CGTTGATTAAGTCCCTGCCCT-3′	5′-TCAAGTTCGACCGTCTTCTCA-3′
OCT-4	5′-GGCCCGAAAGAGAAAGCG-3′	5′-ACCCAGCAGCCTCAAAATCCTC-3′
NCAM	5′-TGGCGAGCAGGATGCGACC-3′	5′-GGCATGGCAACGCACCACCA-3′
TH	5′-CCACAGGCCAAGGGCTTCCG-3′	5′-GGCACCTACCTGCCCTCTTACCA-3′
GFAP	5′-AAGGACGAGATGGCCCGCCA-3′	5′-TGGTGTCCAGGCTGGTTTCTCGA-3′
AFP	5′-AGCAGCTTGTTAAATCAACATGCA-3′	5′-ATTAACTTTGGTAAACTTTCTGACTCAGT-3′
AMYLASE	5′-ACCTCAACAGGTCAGAGATTGTCGT-3′	5′-TCCAGGCCACATGAGCTTGGA-3′
SOX17	5′-CCTCGGGGCATCTCAGTGCCTCA-3′	5′-CTCGGCGTTGTGCAGGTCTGG-3′
PECAM	5′-GCTGAGCGAGTCATGGCCCG-3′	5′-CGCGAAGCACTGCAGGGTCA-3′
DESMIN	5′-CCATGAGCCAGGCCTACTCGTCCAG-3′	5′-AACTCAGCGGGGAGCCGAGTG-3′
SCL	5′-TGGGTGGTCTGGGCTCAGGG-3′	5′-GCTCTGGTGTCAGGTCCTCATCG-3′
PAX6	5′-TTTGCCCGAGAAAGACTAGC-3′	5′-CATTTGGCCCTTCGATTAGA-3′
SOX2	5′-ATGATGGAGACGGAGCTGAA-3′	5′-GGGCTGTTTTTCTGGTTGC-3′
SIX3	5′-CCCACACAAGTAGGCAACTG-3′	5′-GTCCAATGGCCTGGTGCT-3′
CRYAB	5′-GTTCTTCGGAGAGCACCTGTT-3′	5′-GAGAGTCCAGTGTCAAACCAG-3′
CRYAA	5′-AAGGTGCAGGACGACTTTGT-3′	5′-GTGGAACTCACGGGAAATGT-3′
BFSP1	5′-CTCCAGCATCCATTGTGACGA-3′	5′-CGCAGTGTCTCAATCTGCTC-3′
MIP	5′-GTGCTGTATAGCGTTACCCCA-3′	5′-CTCGTCGTATGTGGCAAAGAT-3′
β-actin	5′-CGCACCACTGGCATTGTCAT-3′	5′-TTCTCCTTGATGTCACGCAC-3′

### Immunocytochemistry

The cultured cells were fixed in situ in 4% paraformaldehyde (PFA) for 30 min. Following 3 washes with PBS, the cells were permeabilized in 0.2% Triton X-100 for 30 min, blocked for 1 hour in 10% FCS and 1% BSA in PBS and incubated overnight at 4°C with primary antibodies against NANOG, OCT-4, SSEA-3, SSEA-4, TRA-60, TRA-81, PAX6, α-crystallin andβ-crystallin (Chemicon, USA, 1∶200) in 1% BSA in PBS. On the following day, the cells were washed 3 times with PBS and incubated with fluorescence-conjugated secondary antibodies (anti-rabbit Cy3, Jackson ImmunoResearch, USA, 1∶200) and DAPI in 1% BSA in PBS for 1 h at room temperature. Images were obtained with an inverted Olympus IX81 microscope (Olympus, Tokyo, Japan).

### Western Blotting

Lenses from a 22-yr-old male, 44-yr-old male and 65-yr-old male were thawed on ice and homogenized in PBS, pH 7.2. The homogenates were centrifuged at 5000 rpm for 5 min, and the soluble portion of the lens was retained. Cells were harvested into 1 ml of lysis buffer (50 mM Tris, pH 8.0, 150 mM NaCl, 1% (w/v) Triton X-100 (Sigma, St. Louis, MO, USA), 0.1% (w/v) SDS supplemented with a cocktail of protease inhibitors (Roche Diagnostics, Burgess Hill, UK). Each lane was loaded with 20 mg of total protein. The gels were blotted onto a nitrocellulose membrane (Bio-Rad) and incubated with the following antibodies overnight at 4°C: anti-human PAX6 (Chemicon, USA, 1∶1000), anti-connexin-43 (Santa Cruz, USA, 1∶1000), anti-fibronectin (Santa Cruz, USA, 1∶1000), anti-CRYAB (Chemicon, USA, 1∶1000), anti-CRYAA (Chemicon, USA, 1∶1000) and anti-β-actin (Abcam, USA, 1∶1000). The blots were then incubated for 1 hour with secondary anti-rabbit antibody conjugated to HRP (Santa Cruz, USA, 1∶2000). The membranes were developed using the ECL western blotting detection system (Amersham, UK) according to the manufacturer's protocol.

### Whole-genome expression analysis

A total cDNA microarray was performed using a HG-U133-2 array (Affymetrix) at the Shanghai microarray core. Invariant set normalization was used to normalize arrays at the probe level, and the model-based method was used to calculate expression values. A 20% presence call was used to filter the genes for clustering, resulting in 20,001 probe sets representing individual genes. A hierarchical clustering analysis was performed to distinguish arrays with similar expression patterns.

### Teratoma assay

To test for teratoma formation, iPS cell lines were injected into nude mice. Ethical approval was obtained prior to the commencement of the study from the Council on Animal Care and the Animal Use Subcommittee at the Eye and ENT Hospital of Fudan University (ID: KY2010-68). The “Principles of Laboratory Animal Care” were followed, and every effort was made to minimize the suffering and number of animals used. Briefly, 1×10^6^ iPS cells in 50 µL of ES cell medium were intradermally injected into the mice. The mice were subsequently monitored for teratoma formation and euthanized 6–12 weeks following the injection. The collected teratomas were analyzed using hematoxylin and eosin staining.

### Statistical analysis

All statistical analyses were performed with SPSS for Windows (ver 13.0, SPSS, Inc, Chicago, IL, USA). The results are shown as the mean±standard deviation (SD) or the standard error of the mean (SEM), as indicated. The values obtained from the stem cell array were analyzed using the Pearson correlation coefficient as a measure of similarity. The remaining statistical analyses were performed using an unpaired two-tailed Student's t-tests. P-values<0.05 were considered to be statistically significant.

## Results

### Derivation of iPSCs from HLECs

**Figure 1:**
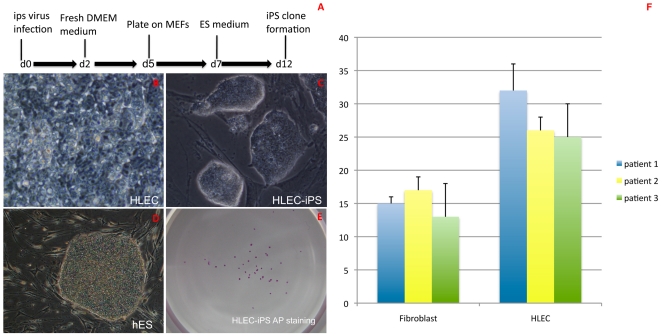
The derivation of induced pluripotent cells from HLECs. HLECs were infected with lentiviruses carrying DNA constructs encoding KLF-4, OCT-4 and SOX-2 to generate induced pluripotent stem cells (HLEC-iPS). (A) A schematic representation of the experimental strategy used to reprogram HLECs. (B) The infected HLECs were plated onto mouse embryonic fibroblasts (MEFs). Colonies with an ES cell-like morphology (C), which appeared as early as 12 days following infection, and (D) H9 ES cell lines. (E) HLEC-iPSCs express pluripotency markers alkaline phosphatase (AP). (F) HLECs can be induced to iPSCs with high efficiency compared with human fibroblast cells: HLEC and human fibroblast cells were collected from three age-related cataract patients. After the iPS factors had been added for 14 days, AP staining was performed, revealing approximately 25 AP-positive clones from 1×10^6^ HLEC cells and approximately 15 clones formed from 1×10^6^ human fibroblast cells.

To identify a strategy for generating iPSCs from HLECs, the cultured HLECs from the cataract patient were infected with lentiviruses carrying DNA constructs encoding KLF-4, OCT-4 and SOX-2 to generate induced pluripotent stem cells (HLEC-iPSCs). This was a 56-year-old male patient diagnosed as age-related cataract and there is no role of traumatic, metabolic or genetic factors influencing the generation and differentiation of this patient's iPSCs. A schematic representation of the experimental strategy used to reprogram the HLECs is shown in Fig. 1A. The infected HLECs were plated onto MEFs on the fifth day following infection (Fig. 1B). Colonies with an ES cell-like morphology formed 12 days following infection (Fig. 1C). The human H9 ES cell line was used as a positive control for pluripotent stem cells (Fig. 1D). These HLEC-iPSCs were labeled with the pluripotency marker alkaline phosphatase (AP) (Fig. 1E). To compare iPS clone formation efficiency, HLEC and human fibroblast cells were collected from three age-related cataract patients. After the iPS factors had been added for 14 days, AP staining was performed, revealing approximately 25 AP-positive clones from 1×10^6^ HLEC cells and approximately 15 clones formed from 1×10^6^ human fibroblast cells (Fig. 1F).

These results demonstrate that iPS clones were successfully induced from the HLECs of the cataract patient with high reprogramming efficiencies. HLEC-derived iPSCs colonies were AP-positive and similar to human ES cells in terms of morphology.

### HLEC-iPS cells share key expression markers and morphology with hESCs

**Figure 2:**
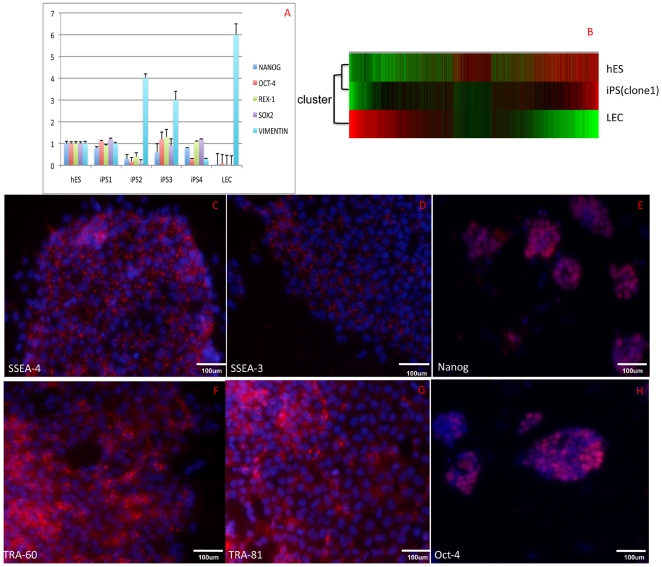
HLEC-iPSCs exhibit key HESC markers and HESC morphology. (A) Real-time RT-PCR analysis of ES markers in HLEC-iPS clones and H9 ES clones. Primers were used that specifically recognize endogenous HESC-specific genes in iPS clones (OCT-4, NANOG, REX-1, SOX-2) and vimentin, which is an HLEC-specific gene. (B) A microarray analysis was performed to compare the gene expression profiles of HLEC-iPSCs, a human H9 ES cell line and primary HLECs. A hierarchical cluster analysis of 8030 orthologous human genes was performed based on the signal ratios. The distances between the genetic profiles of the samples are shown. (C-H): Pluripotency marker staining (red) of the iPS1 clone, (C) SSEA-4, (D) SSEA-3, (E) NANOG, (F) TRA-60, (G) TRA-81 and (H) OCT-4.

We randomly selected four clones from the induced cells (named iPS1, iPS2, iPS3 and iPS4) origining from the 56-year-old age-related cataract patient and compared the expression of key markers and morphology between the HLEC-iPS clones and H9 human ES cell lines. The expression of ES cell markers was examined in iPS and H9 ES clones using real-time PCR analysis with primers targeting endogenous HESC-specific genes in the iPS clones (OCT-4, NANOG, REX-1 and SOX-2) and vimentin, which is an HLEC-specific gene. The RT-PCR results revealed that the gene expression patterns of the iPS1 clone were largely similar to those of hESCs (Fig. 2A). A genome-wide microarray analysis was performed to compare the gene expression pattern of HLEC-iPSCs, H9 ESCs and primary HLECs (Fig. 2B). A hierarchical cluster analysis of 8030 orthologous human genes was performed based on the signal ratios between the different cell types (Fig. 2B). We also stained the iPS1 clone for the pluripotency markers SSEA-4 (Fig. 2C), SSEA-3 (Fig. 2D), NANOG (Fig. 2E), TRA-60 (Fig. 2F), TRA-81 (Fig. 2G) and OCT-4 (Fig. 2H). We observed that these ES markers were highly expressed in the iPS1 clone. These data provide strong evidence that the HLEC-derived iPS colonies were indistinguishable from human ES cells based on the analysis of key markers, and the gene pattern of HLEC-iPSCs was similar to that of ES cells but not that of primary HLECs. The immunohistochemistry (ICH) results provide compelling evidence that iPSCs share the majority of the promising ES cell characteristics.

### iPSCs are pluripotent and can differentiate into three germ layers

**Figure 3:**
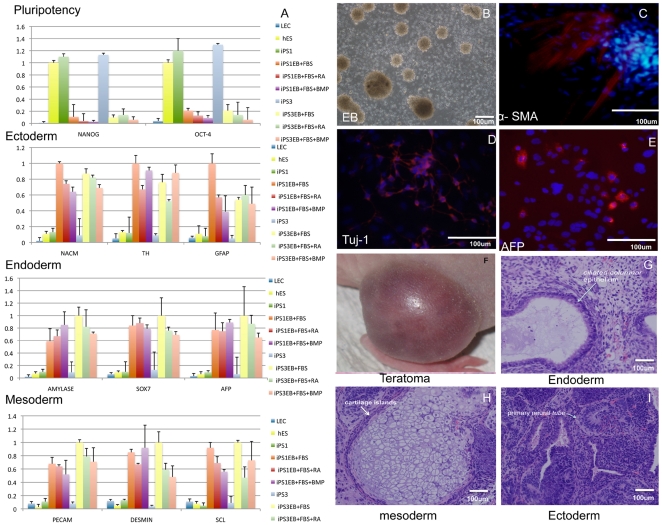
The pluripotency of HLEC-iPS cells and up-regulation of ectodermal, endodermal, and mesodermal markers. (A) Real-time RT-PCR analyses of pluripotency gene expression levels in iPS and control HESCs (H9) induced to differentiate via embryoid body (EB) formation and subsequent plating under the indicated conditions (BMP4, FBS, and retinoic acid (RA)). The results were determined relative to GAPDH expression. The y-axis represents the relative fold change upon differentiation. The expression of marker genes for different germ layers was analyzed. The specificity of each marker for a given germ layer is indicated. (B) Phase-contrast images of EBs generated from iPS1 clones plated onto adherent tissue culture dishes. (C–E) EB-dissociated cells stained by immunofluorescence for mesodermal (C: α-smooth muscle actin (α- SMA)), ectodermal (D: Tuj-1), or endodermal (E: AFP) markers representing each embryonic germ layer. Hematoxylin and eosin staining of teratoma sections from iPSC-derived teratomas is shown (F–I). (F) human iPSCs formed teratomas. (G) ciliated columnar epithelium (endoderm). (H) Cartilage islands (mesoderm). (I) primary neural tube (ectoderm).

We next assessed the pluripotency of iPSCs and their ability to generate all embryonic germ layers. Real-time RT-PCR analysis was performed to assess the expression of pluripotency genes in HLEC-iPSCs clones (iPS1) and ESCs induced to differentiate through embryoid body (EB)formation and subsequent plating under the indicated conditions (BMP4, FBS, and retinoic acid (RA)). The expression of these genes was determined relative to GAPDH expression (Fig. 3A). EB differentiation resulted in the downregulation of pluripotency markers OCT4 and NANOG. The expression of marker genes for different germ layers was also analyzed. The ectodermal markers examined were NACM, TH, and GFAP; the endodermal markers were amylase, SOX7, and AFP; and the mesodermal markers were PECAM, desmin, and SCL. Although the degree of induction for these lineage markers varied somewhat between HESC and iPS clones, the pattern was consistent. For the immunofluorescence experiments, EBs were generated by plating iPS1 clones onto adherent tissue culture dishes (Fig. 3B). After 3 days, the dissociated EB cells were stained by immunofluorescence for mesodermal (Fig. 3C: α-smooth muscle actin (α-SMA)), ectodermal (Fig. 3D: Tuj-1), and endodermal (Fig. 3E: AFP) markers representing each embryonic germ layer. Additionally, following transplantation into nude mice, human iPSCs formed teratomas (Fig. 3F) consisting of all three germ layers, including ciliated columnar epithelium (endoderm, Fig. 3G), cartilage islands (mesoderm, Fig. 3H) and primary neural tube (ectoderm, Fig. 3I). Taken together, these results suggest that typical HLEC-iPS cells exhibit the characteristic morphology and key signaling responses of conventional HESCs and can be differentiated into all of the germ lines.

### iPSCs were efficiently induced to express lens progenitor cell markers using the defined chemical factors

**Figure 4:**
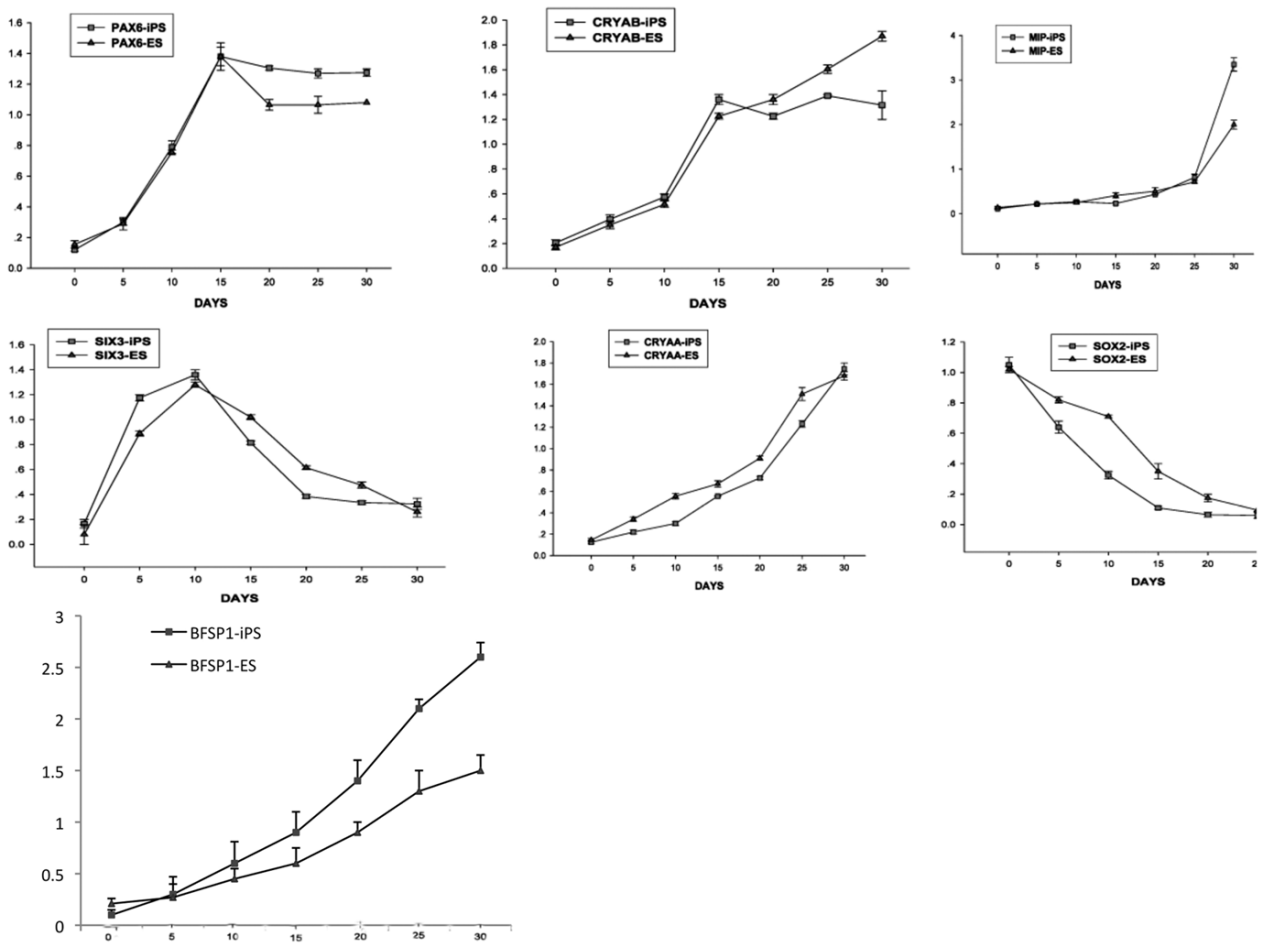
HLEC-iPS can be highly efficiently induced to express lens progenitor cell markers with defined chemical factors. Real-time RT-PCR analysis of 7 lens differentiation markers (PAX6, SOX2, SIX3, CRYAB, CRYAA, BFSP1, and MIP) in iPSCs and ESCs from d 0 to 30 following the initial plating. The results were calculated relative to the average Ct value of GAPDH. For details on the 3-step induction procedure, see Materials and Methods.

**Figure 5:**
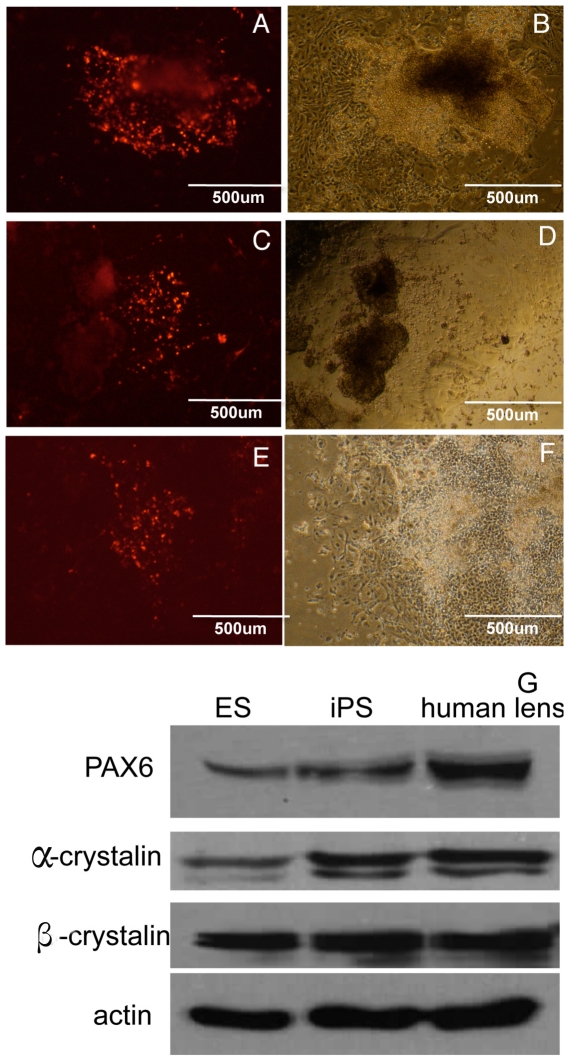
HLEC-iPSCs can be highly efficiently induced to express mature lens cell markers during initial differentiation. The expression of PAX6, α-crystallins and β-crystallins in HLEC-iPSCs differentiated into lens progenitor cells at d 10 following infection is shown. The immunofluorescent detection of PAX6 (red, A, B) is shown, α- crystallin (red, C, D) andβ-crystallin proteins (red, E, F). (G) Western blot analysis of protein expression during the differentiation of HLEC-iPSCs. Specific antibodies were used to detect expression of PAX6, α-crystallin and β-crystallin. Expression of actin is shown as a loading control.

Our next goal was to determine whether these HLEC-derived iPSCs could be further differentiated into lens progenitor cells. To achieve lens-specific differentiation, we applied a 3-step induction procedure, as reported by Yang et al [6]. We also analyzed 7 lens progenitor and differentiation markers (PAX6, SOX2, SIX3, CRYAB, CRYAA, BFSP1 and MIP) in HLEC-iPSCs and hESCs from d 0 to d 30 (Fig. 4). The results indicated that several markers (PAX6, BFSP1 and MIP) were upregulated in HLEC-iPSCs more rapidly than in hESCs. However, the expression of the hESCs marker SOX2 decreased rapidly in the HLEC-iPSCs. The immunocytochemical analyses confirmed that the HLEC-iPSCs have the capacity to differentiate into lens progenitor and primary lens fibroblast cells using a 3-step induction procedure. This finding was determined by the expression of PAX6 (Fig. 5 A, B) α- crystallin (Fig. 5 C, D) and β-crystallin proteins (Fig. 5 E, F). By using western blots to detect the expression levels of these markers, we found comparable levels of these proteins in human lens and iPS-induced cells (Fig. 5 G).From these data, we conclude that this 3-step induction procedure is highly efficient for generating lens cells from patient-specific iPSCs.

### HLE-iPS-derived lens cells display reduced EMT

**Figure 6:**
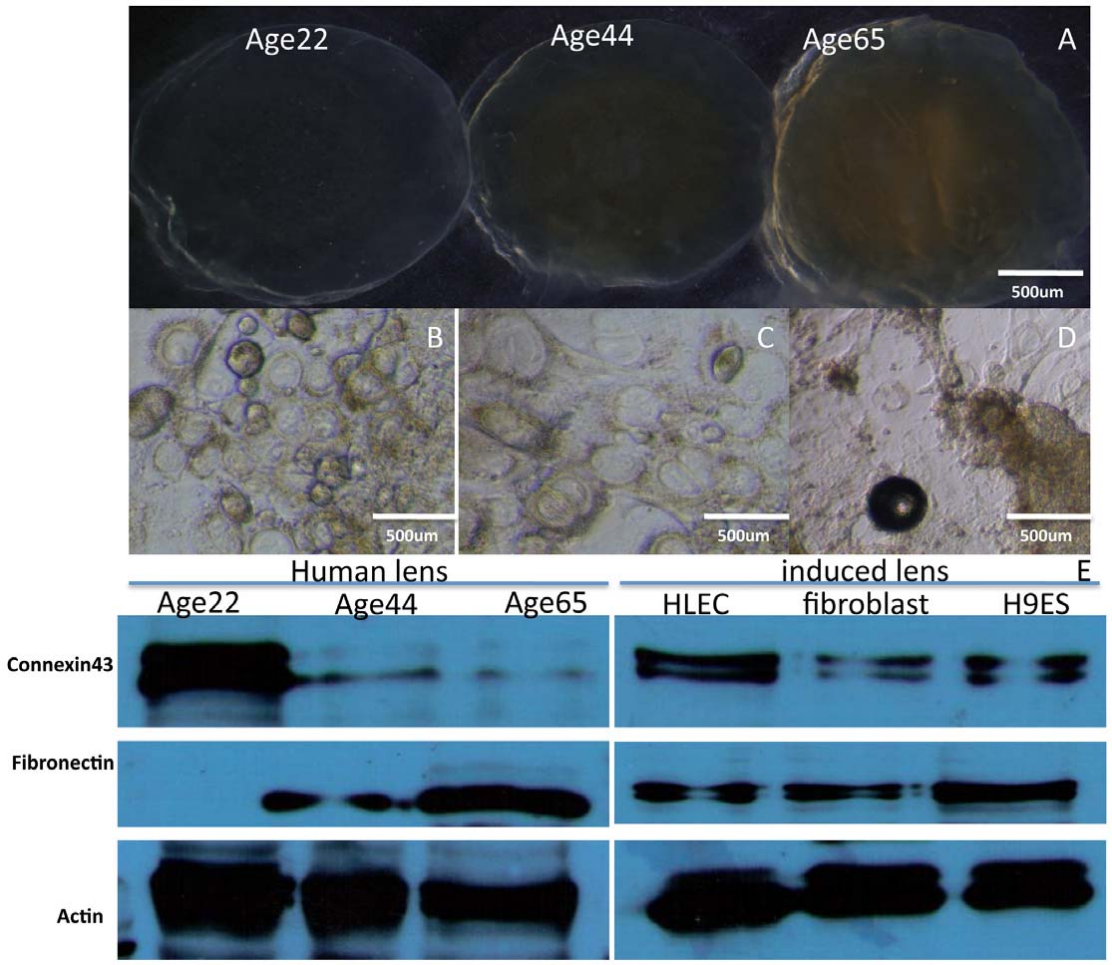
HLEC-derived lens cells exhibit characteristics of young lens cells with reduced EMT. Human lenses at different ages are shown in Fig. 6A, and the morphology of the induced lenses are shown in Fig. 6B–D: (B) the HLEC-iPS-derived lens, (C) human fibroblast-derived lens, (D) h9ES cell line-derived lens. The expression of EMT markers was revealed by western blot. Connexin-43 was abundantly expressed in younger lens cells and HLEC-derived lens cells, while it was significantly suppressed in older lens, skin fibroblast and ES-derived lens cells. In contrast, the expression of fibronectin was significantly increased in older lens, fibroblast and ES-derived lens cells (Fig. 6E).

EMT is the major cause of posterior capsule opacification (PCO) after cataract surgery, a phenotypic conversion characterized by the sequential loss of epithelial markers (e.g., connexin-43) and acquisition of mesenchymal cell markers (e.g., fibronectin). Figure 6 shows a comparison between the human lens at different ages (Fig. 6A) and the iPS- and ES-induced lenses (Fig. 6B–D: B, the HLEC-iPS-derived lens; C, human fibroblast-iPS-derived lens; D, H9 ES cell line-derived lens). The expression of EMT markers was analyzed by western blot. Connexin-43 was abundantly expressed in the younger lens and HLEC-derived lens cells, and it was significantly suppressed in the old lens, skin fibroblast and ES-derived lens cells. In contrast, the expression of fibronectin was significantly increased in the old lens, skin fibroblast and ES-derived lens cells (Fig. 6E). These results show that the ES and fibroblast-iPS-derived lens cells exhibited increased EMT; however the EMT rate in HLE-iPS-derived lens cells was similar to that of normal lens cells.

## Discussion

This study describes a highly efficient procedure for generating lens cells from patient-specific iPSCs. The ability of different hESC and iPSC lines to differentiate into specific cell types has been shown to vary by cell line. For example, foreskin iPS lines spontaneously differentiate into retinal pigment epithelium (RPE) cells at a lower rate than iPS lines from the eye [17]. The reason for this difference is unclear, but it may contribute to the decision of which iPS lines are used for research and treatment applications. In the present study, we showed that the HLEC-derived iPS (HLEC-iPS) cell colonies were indistinguishable from human ES cells with respect to their morphology, gene expression profile, expression of pluripotent markers and ability to generate all three embryonic germ layers in vitro and in vivo. HLEC-iPS can be highly and efficiently differentiated into large numbers of lens progenitor cells through the use of defined factors (Noggin, BMP and FGF2). The expression of lens-specific markers was induced and maintained in these cells.

Lens research aims to gain insights into the cellular and molecular basis of ophthalmological disease. Furthermore, the lens serves as a valuable experimental system with which to address fundamental biological questions. The lens is an encapsulated avascular organ that focus light onto the retina. Millions of individuals are blinded or exhibit visual impairment, due to pathologies of the lens [18], and cataracts are among the leading causes of blindness in elderly populations [1]. The distinct architecture of the lens is established early in life and maintained as the lens continues to grow [4]. This growth depends on the controlled proliferation of lens epithelial cells and their precise local differentiation into long and transparent fiber cells. Any disruption to these normal cellular processes can disrupt lens architecture, resulting in cataract formation [4]. Currently, surgery is often the only successful treatment, although this approach it is not without later complications that may lead to what is termed secondary cataract [19]. A common complication following surgery results from the aberrant behavior of the remaining lens cells. These residual cells transform into pathological cell types, which can impair vision yet again [20]. Current cataract research primarily aims to identify the molecules and mechanisms involved in the regulation of normal lens cell behavior. Cataract prevention is also a vital topic in preventative medicine and offers hope to the multitude of individuals worldwide who cannot be aided by intraocular lens implantation. The patient-specific iPSCs described here will open new avenues of research to study the molecular mechanisms of patient-specific lens development. This technique will also advance the study of iPS-derived lens cells isolated from cataract patients. Because lens formation is critical to eye development, this study will also aid in describing human embryonic lens development, aging and related diseases.

Clearly, iPS technology provides a new method for establishing a patient-specific model of lens development and disease. In 1901, Hans Spemann introduced the concept of inductive interactions by studying lens development [21]. Induction is a process by which one group of cells or tissue regulates the development of a separate cell population or tissue. Since the introduction of this concept, several studies have sought to better understand the role of specific signaling molecules, the interplay between different signals and tissue interactions in regulating lens induction and patterning events. This knowledge is not only important for understanding normal lens development but also key to defining the general mechanisms of cell specification and gaining a better understanding of lens function and pathology. There is therefore a great need for patient-specific disease models in clinical research. In our work, we attempted to generate patient-specific lens cells using iPS technology. The iPS cells share the majority of the therapeutic and research potential of ESCs but lack the ethical and political complications, as well as the immunological considerations [5]. These cells are also more clinically relevant for many applications and are therefore an excellent choice for research in toxicology, regenerative medicine and other fields. Pluripotent stem cells can be generated by reprogramming patient somatic cells, and patient-tailored iPS cells will be valuable for regenerative medicine [6], [22], [23]. However, several obstacles must be overcome before therapeutic applications of iPS cells for human diseases can be implemented. One issue is the question of how to directly differentiate iPS cells into specific donor cells. Other challenges to using iPSCs include determining how to reprogram primary cells from patients with diseases and showing that these reprogrammed cells can be differentiated into cell types that function properly upon transplantation into disease treatment models. Overcoming these challenges may allow general human cell reprogramming through the expression of defined factor to be an applicable therapeutic approach.

Our data demonstrate that the transduction of defined factors (OCT-4, SOX-2 and KLF-4) into patient-specific HLECs leads to the formation of iPSCs with high reprogramming efficiencies. Our analysis indicates that one iPS clone, iPS2, was only partially reprogrammed to an ES cell state based on gene expression profiles and the inability to form EBs. The iPSCs we generated via the expression of defined factors in HLECs were highly similar to HESCs in terms of both morphology and physiology. Next, we use small molecules to induce the lens-specific differentiation of the patient-specific iPSC. Multiple signal transduction pathways, including FGF, TGF-β and Wnt, have been implicated in lens development [3]. Some of these factors, such as FGF, have been shown to have the same cell-growth promoting effect in an iPS system [6]. Our results indicate that some markers (PAX6, BFSP1 and MIP) can be upregulated in differentiated HLEC-iPS cells more rapidly than in hESCs; however, expression of the hESCs marker SOX-2 decreased rapidly in the HLEC-iPSCs. PAX6 is a key regulatory gene throughout lens development [23]. Our technique for lens cell differentiation increased and maintained PAX6, CRYAB and CRYAA expression, which was similar to the effect observed in Yang's study [6].

In vertebrates, lens progenitor cells originate from the preplacodal region (PPR) [24], [25]. PPR cells subsequently give rise to other cell lineages, including the anterior pituitary, olfactory epithelium, and inner ear [26]. The adult lens has a relatively simple structure and is composed solely of epithelial and fiber cells. The lens's isolation from nerves and blood vessels in the adult makes it a tractable model for investigating the mechanism of epithelial cell regulation. Multiple signal transduction pathways, including FGF, TGF-βand Wnt, have been implicated in lens development [27], [28]. These studies have suggested that FGFs, BMP4, BMP7, Noggin and Wnts might be excellent candidates for lens cell differentiation from human ES cell cultures [29], [30], [31], [32], [33]. Our results showed HLEC-derived iPS cells can be highly efficiently induced to express lens progenitor and primary lens fibroblast cell markers through the use of defined chemical factors. The elevated expression of some markers (PAX6, BFSP1, and MIP) can be detected when compared with lens cells induced from H9 ES cells. We detected the expression of a number of critical genes for lens development and maturation in patient-specific iPSCs after differentiation and obtained similar results to what was observed for hESC differentiation. In our future work, we will attempt to define the induction conditions and assay lens architecture and function.

Approximately 20–25% of patients undergoing cataract surgery experience PCO within 2 years [34], [35]. Emerging evidence suggests that LECs can undergo an EMT that bears morphological and molecular resemblance to forms of PCO [36], [37]. A hallmark of EMT in LECs is the loss of epithelial cell-cell gap junctions caused by the downregulation of connexin-43 [38]. The connexins are important for both the formation of cell-cell channels, allowing the transmission of functional molecules, and the expression of genes encoding functional proteins [39], [40]. In our study, extensive EMT was observed in both older individuals and the ES/fibroblast-iPS-derived lens cells, while this transaction was relatively low in the HLEC-iPS-derived cells, suggesting that the HLEC-iPS-derived lens cells were more suitable for lens regeneration than the ES/fibroblast-iPS cells.

Cataracts are a common human disease often observed in animal models as well. Over the last several decades, considerable progress has been made in identifying and characterizing many of the molecules involved in normal lens biology and its pathology. Much of this research has been made possible through the establishment and use of lens models. However, a systematic approach for studying human cataracts has been hampered by the lack of appropriate human-derived models. Moreover, because of the small size of the families available for detailed genetic investigations, it is necessary to develop appropriate human-derived models to identify the genes responsible for cataract formation and to analyze the mechanisms leading to lens opacification. There are several advantages of using patient-specific HLEC-derived iPSCs regarding efficiency, convenience and application value. First, several studies have demonstrated that cells from the target tissue are more easily differentiated back to their original source than other cells [17]. Therefore, iPSCs from primary human lens epithelial cells should more efficiently differentiate into lens cells than those from skin fibroblasts. We compared the clone formation efficiency between HLECs and fibroblasts and found that HLEC–derived iPSCs had higher efficiency. Second, we found that fibroblast and human ES-derived iPSCs could undergo an EMT during the chemically-induced differentiation, which bears a morphological and molecular resemblance to forms of PCO. However, we detected reduced levels of EMT markers during HLEC-derived iPSC differentiation, suggesting that HLEC-iPS may be more suitable for clinical applications. Third, the piece of the anterior capsule with the LECs attached to it was obtained by capsulotomy during cataract surgery. This approach is a simple, convenient and safe way to obtain primary cells. Based on our results, there was no requirement to obtain patients' skin fibroblasts through a second method. Through this patient-specific model, we will be able to further study how to inhibit cataract formation and better understand the pathogenetics of congenital cataracts as well as developmental lens defects, such as lenticular coloboma and spherophakia. This human-derived model will serve as a powerful tool to further our understanding of lens biology and pathology, and there is no doubt that it will continue to serve in such a capacity as new developments are realized, and putative treatments for aberrant lens cell behavior are tested.

HLECs have generally been discarded in cataract operations. In our study, we found that these cells were useful materials for the production of cataract patient-specific iPSCs. These iPSCs can be efficiently induced to differentiate into lens cells using defined chemical conditions. Our research revealed that iPS cells that are induced by OCT-4, SOX-2, and KLF-4 inherently express lens-specific markers, indicating that these cells are valuable for lens regeneration research. In this study, we present a technique for reprogramming HLECs to pluripotent stem cells and the direct differentiation of these cells into lens progenitor cells, thereby artificially directing cell fate through the use of transcription factors. It is necessary to study iPS lines from multiple patients and compare these different iPS lines with different ES cell lines. We will further investigate this human-derived model, thoroughly characterize it and put it into effect in our future research. In conclusion, we present a preliminary step towards the goal of patient-specific model construction and lens regeneration based on iPS technology and a three-step induction procedure. These findings demonstrate the usefulness of this model for studying the molecular mechanisms of human lens development and the biology of lens cells differentiated from cataract patient-derived iPSCs.
